# Protection by hydroxychloroquine prevents placental injury in obstetric antiphospholipid syndrome

**DOI:** 10.1111/jcmm.17459

**Published:** 2022-06-29

**Authors:** Jing Liu, Liting Zhang, Yijia Tian, Shuting Wan, Min Hu, Shasha Song, Meihua Zhang, Qian Zhou, Yu Xia, Xietong Wang

**Affiliations:** ^1^ Department of Obstetrics and Gynaecology, Shandong Provincial Hospital Shandong University Jinan China; ^2^ Maternal and Child Health Care Hospital of Shandong Province Jinan China; ^3^ The Laboratory of Placenta‐Related Diseases Key Laboratory of Birth Regulation and Control Technology of National Health and Family Planning Commission of China Jinan China; ^4^ Central Laboratory Shandong Provincial Hospital Affiliated to Shandong First Medical University Jinan China

**Keywords:** animal model, antiphospholipid antibody, APS, hydroxychloroquine, obstetric antiphospholipid syndrome, trophoblast

## Abstract

Obstetric antiphospholipid syndrome (OAPS) is mediated by antiphospholipid antibodies (aPLs, and anti‐β2 glycoprotein I antibody is the main pathogenic antibody), and recurrent abortion, preeclampsia, foetal growth restriction and other placental diseases are the main clinical characteristics of placental pathological pregnancy. It is a disease that seriously threatens the health of pregnant women. Hydroxychloroquine (HCQ) was originally used as an anti‐malaria drug and has now shown benefit in refractory OAPS where conventional treatment has failed, with the expectation of providing protective clinical benefits for both the mother and foetus. However, its efficacy and mechanism of action are still unclear. After clinical data were collected to determine the therapeutic effect, human trophoblast cells in early pregnancy were prepared and treated with aPL. After the addition of HCQ, the proliferation, invasion, migration and tubule formation of the trophoblast cells were observed so that the therapeutic mechanism of HCQ on trophoblast cells could be determined. By establishing an obstetric APS mouse model similar to the clinical situation, we were able to detect the therapeutic effect of HCQ on pathological pregnancy. The normal function of trophoblast cells is affected by aPL. Antibodies reduce the ability of trophoblast cells to invade and migrate and can impair tubule formation, which are closely related to placental insufficiency. HCQ can partially reverse these side effects. In the OAPS mouse model, we found that HCQ prevented foetal death and reduced the incidence of pathological pregnancy. Therefore, HCQ can improve pregnancy outcomes and reverse the aPL inhibition of trophoblast disease. In OAPS, the use of HCQ needs to be seriously considered.

## 1 INTRODUCTION

Antiphospholipid syndrome (APS) is a systemic autoimmune disease, with thrombosis and/or pathological pregnancy as the main clinical features. APS can occur alone or can coexist with other autoimmune diseases.^1^ The main clinical feature of pathological pregnancy, such as stillbirth, severe preeclampsia, spontaneous abortion, and foetal growth restriction, is obstetric APS (obstetric antiphospholipid syndrome, OAPS).^2^ The pregnancy complications of OAPS are believed to affect the function of the placenta, damaging placental decidua cells and trophoblast cells, affecting placental implantation, material transport, and in severe cases, causing thrombosis and infarction in the placenta. It is a systemic autoimmune disease with or without thrombosis that seriously threatens the health of pregnant women and their foetuses. The pathogenesis of OAPS is not yet clear, the clinical heterogeneity is high, and there are many bottlenecks in diagnosis and treatment.^3^, ^4^, ^5^

Anti‐phospholipid antibodies (aPLs) are pathogenic antibodies to APS and are a heterogeneous group of antibodies against phospholipids and phospholipid‐binding proteins.^6^, ^7^ For patients with suspected OAPS, it is recommended to determine lupus anticoagulant (LA), anticardiolipin antibody (aCL) and anti‐β2 glycoprotein I antibody (anti‐β2GP1 Ab).^1^ In the normal population, the detection rate of aPLs is only 0%–0.5%, while the detection rate in patients with preeclampsia is 7%–17%. The detection rate can reach 5%–20% in patients with recurrent miscarriage. For positive aPLs among women, 15%–30% have foetal growth restriction during pregnancy, and approximately 50% have experienced recurrent miscarriages.^8^, ^9^ In OAPS, aPLs are an important cause of pathological pregnancy.

Among these pathogenic antibodies, β2 glycoprotein I (β2GPI)‐dependent antiphospholipid antibodies are considered to be the main pathogenic autoantibodies in OAPS. The target antigen β2GPI has a different tissue distribution and is highly expressed in trophoblast cells. Anti‐β2GP1 Ab could bind to trophoblasts and decrease hCG secretion.^10^, ^11^ Some experiments have shown that antibodies affect the cell invasion function of trophoblast cells.^12^, ^13^ In in vivo experiments in mice, the antibody entered the blood circulation and rapidly deposited in the foetal sacs, affecting mouse foetal cortical neuron cytoarchitecture.^14^ The antibodies isolated from patients could cause foetal loss.^15^ Polyclonal antibodies, including high concentrations of anti‐β2GP1 Ab infusion in pregnant CD39‐ or CD73‐knockout mice, trigger an increase in miscarriages. This study concludes the possibility that perturbation of CD39 (NTPDase1) and CD73 (nucleotidase), could act as the “second hit” in the manifestation of APS.^16^ In OAPS patients, placental inflammation and thrombosis or infarction are important pathogenic factors of pathological pregnancy. The anti‐β2GP1 Ab can directly or indirectly cause these pathological pregnancy injuries.

Hydroxychloroquine (HCQ) was originally used as an anti‐malaria drug and has now become popular in treating autoimmune rheumatic diseases. It has been shown to be beneficial to mothers and foetuses in some autoimmune diseases.^17^, ^18^ In primary or secondary antiphospholipid syndrome, HCQ has been shown to reduce aPLs titers in the plasma.^19^, ^20^ Prenatal exposure to HCQ in patients with anti‐SSA/SSB seropositivity, could decrease the risk of foetal heart manifestations of neonatal lupus.^21^, ^22^ However, there are few clinical data about the use of HCQ in obstetrical primary APS. HCQ can be used for refractory OAPS where conventional treatment has failed (low dose aspirin and heparin are the first choice for the treatment of OAPS) and is often administered empirically by obstetricians.^23^, ^24^ Due to the lack of clinical randomized clinical trials and the unclear mechanism of action, this cheap and safe drug has not been widely used in OAPS patients.

HCQ was able to mitigate the features of endothelial dysfunction in preeclampsia induced by recombinant TNF‐a in vitro.^25^ HCQ reduced aPL binding to syncytiotrophoblasts and reversed aPL‐mediated disruptions of annexin A5.^26^, ^27^ Some of these in vitro studies demonstrated that HCQ can partially enhance the invasion function of trophoblasts and affect the release of inflammatory factors. It did not alter the release of hCG, sFlt‐1, PlGF or hPL, the levels of IL‐10 were increased, and the secretion of IL‐1β, TNFα, IL‐6 or MCP‐1 did not change.^28^, ^29^ An in vivo study have shown that HCQ protects pregnancy in APS patients by inhibiting complement activation in placentas and foetal brains.^14^ Therefore, how HCQ plays a role in OAPS has not yet been clearly studied.

Accordingly, we systematically evaluated the effect of anti‐β2GP1 antibodies on trophoblast cell function and investigated the effects of HCQ in vivo and in vitro. We aimed to determine whether HCQ can reverse the adverse effects of antibodies on multiple functions of trophoblasts and whether HCQ plays a crucial role in the pathogenesis of adverse pregnancy and foetal outcomes.

## 2 MATERIALS AND METHODS

### 2.1 HCQ treatment on pregnancy outcome

We conducted a retrospective study. The records of all outpatients and inpatients diagnosed with antiphospholipid syndrome in the obstetrics department from July 2019 to December 2020 were searched. All patients met the Sidney Laboratory criteria for APS that was updated in 2006.^30^ Information from the OAPS patients was obtained at Shandong Provincial Hospital affiliated with Shandong University. Our final cohort included 96 OAPS patients with different gestational weeks. The patient antibody titres and other tests were obtained from the work of the laboratory department of Shandong Provincial Hospital.

The patients were divided into two groups: one group received HCQ treatment in this pregnancy, and the other group was not exposed to HCQ. The basic data of the two groups of patients, including the patient's general condition, disease classification, pregnancy history and antibody titers, were recorded. The occurrence of adverse events was recorded and compared between the two groups of patients for this pregnancy.

### 2.2 Cell culture

HTR‐8/SVneo is a first‐trimester human extravillous trophoblast cell line purchased from the American Type Culture Collection (ATCC; Manassas, VA). The cell culture medium included RPMI 1640 medium supplemented with 10% foetal bovine serum (FBS, Gibco) and 100 nmol/L penicillin/streptomycin (Gibco). The cells were all maintained in a cell incubator at 37°C and 5% CO_2_.

### 2.3 The preparation of anti‐β2GP1 antibody

We developed an anti‐β2GP1 Ab in collaboration with Affinity Biosciences. The gene polypeptide sequence was determined using AbDesigner software as Trairak Pisitkun et al. previously described.^31^ The polypeptide sequence is GRTCPKPDDLPF, and the required polypeptide chain was synthesized. Then, we coupled it with the immune antigen. Immunization of Balb/c mice was enhanced, and blood was collected to screen out immunized mice. The mouse spleen was collected and crushed and was mixed with DMEM to form a cell suspension. The SP20 cells and splenic cells were mixed, and then preheated PEG was added to fuse the cells. These cells were resuspended, and the fused cells were screened by ELISA to establish a monoclonal cell line. The fluid from the ascites in the mouse abdominal cavity was taken to identify and purify the antibody and to measure the concentration. Technical route of antibody preparation is shown in Figure S1. Before using the monoclonal antibody, its reactivity was investigated compared with polyclonal antibodies purified from APS patients. There was no significant difference between the two antibodies (Figure S2). All aPL used in the experiment were prepared with an anti‐β2GP1 Ab.

### 2.4 Cell proliferation

Cell proliferation was detected by a Cell Counting Kit‐8 (CCK‐8) assay. HTR‐8/SVneo cells (5 × 10^4^ cells/well) were grown in wells of a 96‐well plate in culture medium and were incubated overnight. After the cells grew enough to adhere to the wall, the cell media was supplemented with different treatments in three groups. The cells were treated with HCQ (Sigma Aldrich) at 0, 0.01, 0.1, 1, 10, and 100 μg/ml. The cells were treated with aPL at 10, 20, 30, 50 μg/ml. The cells were treated with aPL 20 μg/ml with HCQ at 1 and 10 μg/ml. To detect the cell viability, 10 μl CCK‐8 per 100 μl medium was added. After incubation for 2 h, the optical densities were measured at 450 nm. The detection time was 12 h, 24 h, 48 h, 72 h.

### 2.5 Tube formation

Matrigel (BD Bioscience) was diluted 1:1 with serum‐free medium. After dilution, 50 μl was added to each well of the 96‐well plate and the plates were incubated at 37°C for 30 min to solidify the Matrigel. HTR‐8/SVneo cells were planted in a 96‐well plate 4 × 10^4^ cells/well with the culture medium. HCQ (1 μg/ml, 10 μg/ml) and aPL (10, 20, 30, 50 μg/μl) were added to the experimental group, and the plates were incubated for 6 h at 37°C and 5% CO_2_. Tube formation was assessed using an inverted microscope and was analysed by ImageJ software.

### 2.6 Scratch wound‐healing assay

HTR‐8/SVneo cells were evenly seeded in a 6‐well plate (5 × 10^5^) in an incubation room at 37°C and 5% CO_2_ overnight. A 200 μl sterile pipette tip was scratched straight across the confluent monolayer to make a clear and equal‐width scratch in the middle of each well. PBS was used to gently wash off the exfoliated cells. HCQ and aPL were added to the groups, and the dosages were the same as those mentioned above. After 24 h, the cell migration area was observed by inverted microscopy (4 × objective) and was analysed using ImageJ software.

### 2.7 Cell invasion

To assess the cell invasion, a two‐chamber Transwell migration assay was used as previously described. Matrigel (BD Bioscience) was diluted 1:8 and was precoated with 60 μl in a chamber membrane at 37°C overnight. HTR‐8/SVneo trophoblast cells (5 × 10^4^/well in 200 μl RPMI 1640 medium supplemented with 2% FBS and 100 nmol/L penicillin/streptomycin) were seeded in an 8‐μm pore size cell culture insert that served as the top chamber. The lower chambers (24‐well culture plate) were filled with 600 μl of complete medium (RPMI 1640 medium supplemented with 10% FBS and 100 nmol/L penicillin/streptomycin). After a 24 h incubation, the upper chambers were removed, and cotton swabs were used to wipe the noninvaded cells. Trophoblast invasion across the membrane was fixed with 4% paraformaldehyde and was stained with crystal violet dye. The invaded cells were observed by an inverted microscope (Olympus, Tokyo, Japan) at a magnification of 100 ×, and five fields of view were randomly selected. The cell invasion number was estimated using ImageJ software.

### 2.8 Mouse feeding

The animal housing and experimental protocols were performed in compliance with the animal ethics committee of Shandong University, Jinan, China. C57BL/6 mice (6–8 weeks) were purchased from Weitong‐Lihua Experimental Animal Center (Beijing, China). The weight of the mice was approximately 18 g–22 g. All mice were housed under standard environmental conditions (specific pathogen‐free, SPF) with free access to drinking water and a commercial mouse chow diet. The mice were housed at a temperature of 20 ~ 26°C, humidity at 30 ~ 70%, and a 12 h:12 h light/dark cycle. Female mice were mated with previously isolated males and caged at a ratio of 2:1 for 12 h. The presence of a vaginal plug confirmed pregnancy as day 0 of pregnancy (P0).

### 2.9 Mouse model construction of OAPS

The mouse model of OAPS was established by intravenous injection of antibodies. 100 μg/mouse of aPL antibodies were administered on Day 0 and Day 7 of pregnancy and at a dose of 50 μg/mouse on Day 8. This model could maintain a stable antibody concentration in pregnant mice, which is closely consistent with the clinical condition. HCQ was dissolved in sterile distilled water and was administered by a sterile insulin syringe (100 mg/mouse/day) on Day 8–Day 14 in a group of pregnant mice. Based on the clinical medication guidelines, in which the recommended dosage of HCQ is 6.5 mg/kg/day, we used a dose of 100 mg HCQ/mouse/day.^32^ The control groups were treated with sterile saline instead of antibodies and HCQ.

Day15 was the day that pregnant mice were euthanized for dissection. The foetal resorption frequency was (number of foetal loss)/(total number of foetuses and resorptions) × 100%. The uterus was dissected, and then the foetuses and placentas were removed and weighed. The required specimen was removed, washed in sterile PBS to remove the remaining blood, soaked in formalin or stored at −80°C.

### 2.10 Pathological studies

After euthanasia, the placentas were removed and fixed in 4% paraformaldehyde. Low‐concentration to high‐concentration alcohol was used as a dehydrating agent to gradually remove the water in the tissue mass. Then, the alcohol was replaced in the tissue block with xylene before embedding the tissues in paraffin. The embedded paraffin blocks were cut into slices (5–8 μm), were placed on a glass slide, and were dried at 45°C. Before dying, xylene and different concentrations of alcohol were used for deparaffinization. The slides were stained with haematoxylin and eosin for histological analysis.

### 2.11 Statistical analyses

Data are expressed as the mean ± SEM. All data came from three independent experiments. Statistical differences between the groups were assessed using one‐way anova with a subsequent two‐tailed Student's *t*‐test. *p* Values < .05 were considered statistically significant.

## 3 RESULTS

### 3.1 HCQ might ameliorate clinical‐pathological pregnancies of OAPS

We conducted a retrospective study involving 96 patients to analyse whether HCQ has a positive effect on the pregnancy outcome of APS. After analysing the situation of OAPS patients from June 2019 to December 2020, HCQ can be considered to have a positive effect on the prevention of some obstetric complications.

As indicated in Table 1, HCQ was more likely to be used to treat patients with APS + SLE and patients with a history of recurrent miscarriage. No correlation was found between the positive rate of the two antibodies and whether to use HCQ treatment. Table 2 shows the pregnancy outcome with antiphospholipid antibodies in patients who were treated with or without HCQ. In the cases we counted, without using HCQ, the proportion of spontaneous abortions, foetal distress and amniotic fluid muddy increased. On the contrary, in the HCQ group, the incidence of preterm birth increased, and the incidence of miscarriage was low. Although some of them were not statistically significant, we observed that HCQ appeared to be able to reduce some placental complications. Foetal distress in particular, treatment of HCQ remained a protective factor after the removal of confounders. Does this mean that HCQ may play a role in placental pathological pregnancy?

**TABLE 1:** Clinical characteristics of pregnant women with antiphospholipid antibodies enrolled in this study

Characteristic	With HCQ	%	Without HCQ	%	Significance
Patient	59		37		
Age (y)	31		33		
Number of pregnancies	93		46		
*Type of diseases*					
APS only	40	67.8	29	78.4	
SLE and APS	8	13.6	0	0	*p* = .02
APS and other systemic diseases*	11	18.6	8	21.6	
Previous thrombosis	3	5.1	0	0	
*History of pathological pregnancy*					
Spontaneous abortions before the 10th week	39	66.1	16	43.2	*p* = .03
Foetal Loss beyond the 10th week	3	5.1	1	2.7	
Placenta‐mediated complication	9	15.2	6	16.2	
aPLs positivity	59		37		
aCL positivity	27	45.8	21	56.7	
anti‐β2GPI positivity	34	57.6	19	51.4	
LA positivity	26	44.1	22	59.5	

*Note*: APS and other systemic diseases: In our data, iMild sjogren's syndrome or thrombocytopenia were included.

Abbreviations: aCL, anticardiolipin antibody; anti‐β2GPI, anti‐β2 glycoprotein I antibody; aPLs, antiphospholipid antibodies; APS, antiphospholipid syndrome; SLE, systemic lupus erythematosus.

**TABLE 2:** Pregnancy outcomes of APS pregnant women with or without HCQ

	With HCQ	%	Without HCQ	%	Significance
Live births	58	98.3	34	91.9	
aPLs related pregnancy morbidity					
Preterm live births (<37 w)	12	20.3	5	13.5	
Spontaneous abortions before the 10th week	0	0	3	8.1	*p* = .05
Foetal loss beyond the 10th week	1	1.7	0	0	
*Placenta‐mediated complication*					
Preeclampsia	8	13.6	6	16.2	
Abruption placentae	1	1.7	1	2.7	
FGR	4	6.7	2	5.4	
Foetal distress	3	5.1	9	24.4	*p* = .01
Placental hematoma	5	8.5	4	10.8	
Amniotic fluid muddy	0	0	4	10.8	*p* = .02
*Mode of delivery*					
Vaginal delivery	4	6.7	5	13.5	
Caesarean section	54	91.5	32	86.5	
Gestation duration (weeks)	36.2		37.2		
Birth weight	3028 g		2996 g		

Abbreviations: aPLs, antiphospholipid antibodies; FGR, foetal growth restriction.

From these data, we found that antiphospholipid antibodies can cause many placental pathological pregnancies, and HCQ treatment may be effective. To study whether HCQ reverses the harm of antibodies and protects the placenta to reduce the occurrence of abortion, we carried out the following in vitro and in vivo experiments.

### 3.2 The safety profile of HCQ in trophoblast cell proliferation

**FIGURE 1:**
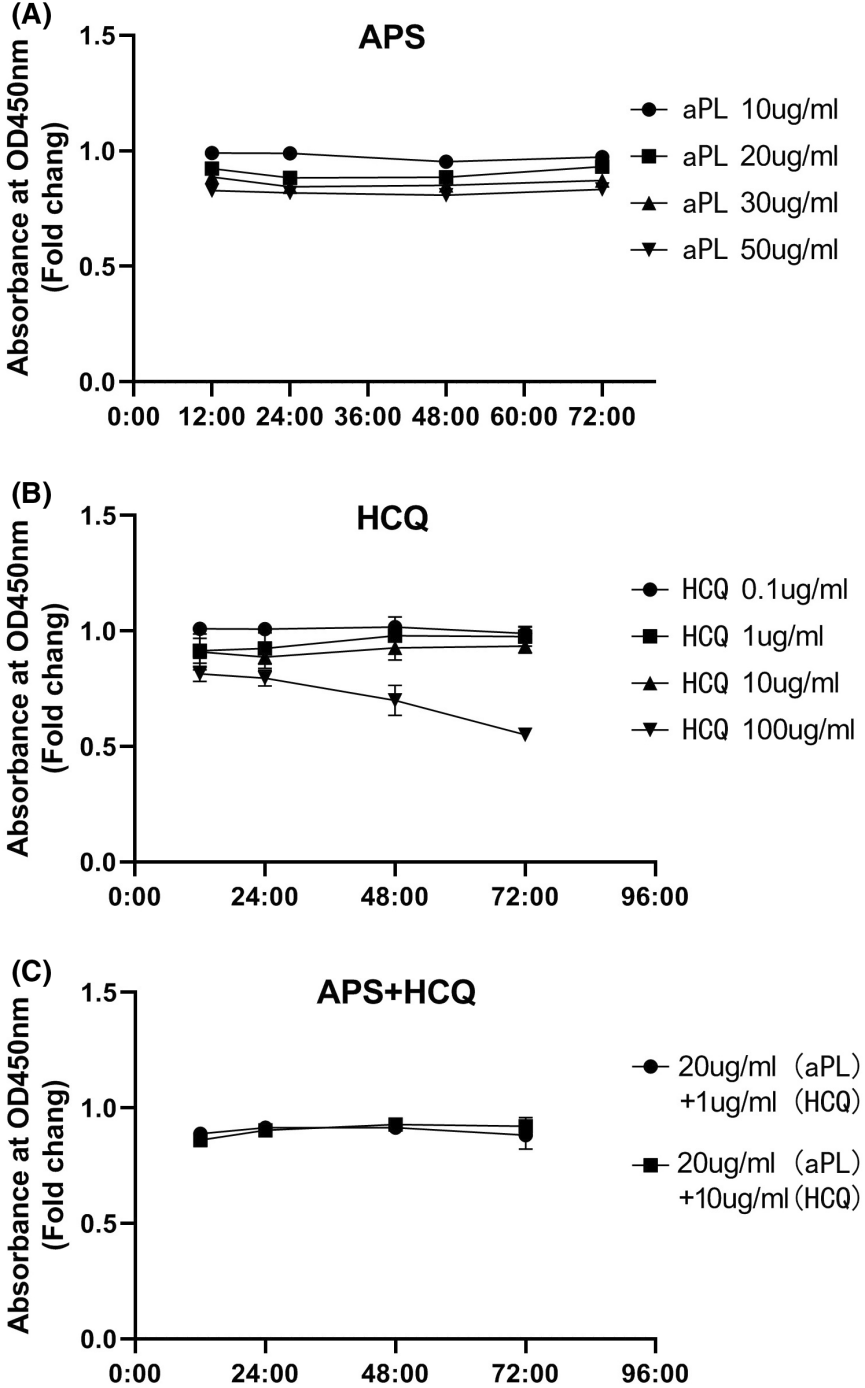
Changes in viability of the first‐trimester trophoblast cell line (HTR8) under the influence of hydroxychloroquine (HCQ)/antiphospholipid antibody (aPL) concentrations at different doses. Cell viability is shown as a percentage of the no treatment control. (A) HTR8 was incubated with no treatment or aPL at 10, 20, 30, 50 μg/ml for 12, 24, 48.72 h. (B) HTR8 was incubated with HCQ at 0, 0.1, 1, 10, or 100 μg/ml for 12, 24, 48.72 h. (C) HTR8 was incubated with no treatment or aPL plus HCQ. The two groups are APS (aPL at 20 μg/ml) plus HCQ (HCQ at 1 μg/ml), and APS + HCQ (aPL at 20 μg/ml plus HCQ at 10 μg/ml) for 12, 24, 48, 72 h. Data are from three independent experiments

The issue of the potential for toxicity by HCQ has been of particular concern for patients with OAPS.^32^ The HTR‐8/SVneo cell viability assay was the first objective to determine the appropriate concentration of HCQ to determine the therapeutic dose to prevent cytotoxicity. Figure 1B shows that 100 μg/ml HCQ significantly reduced cell viability, and 10 μg/ml, 1 μg/ml and 0.1 μg/ml were noncytotoxic doses. Therefore, a concentration of 10 μg/ml were undertaken in all subsequent experiments. The viability of cells was not time‐dependent.

Using the same method, the experiments of trophoblast cell proliferation were determined with a range of aPL concentrations (Figure 1A). When compared to the untreated control, 50 μg/ml was a cytotoxic dose. In the following experiments, we used a concentration of 20 μg/ml, which is consistent with previous studies. There was no significant effect on cell viability at the concentrations of 2 kinds of experimental doses—APS 20 μg/ml + HCQ 10 μg/ml, APS 20 μg/ml + HCQ 10 μg/ml (Figure 1C).

Therefore, at therapeutic doses, HCQ does not affect the growth and proliferation of placental trophoblast cells. We found that the proliferation and activity of placental trophoblast cells were not targets of HCQ treatment of OAPS.

### 3.3 HCQ reverses the adverse effects of aPL on tubule formation

**FIGURE 2:**
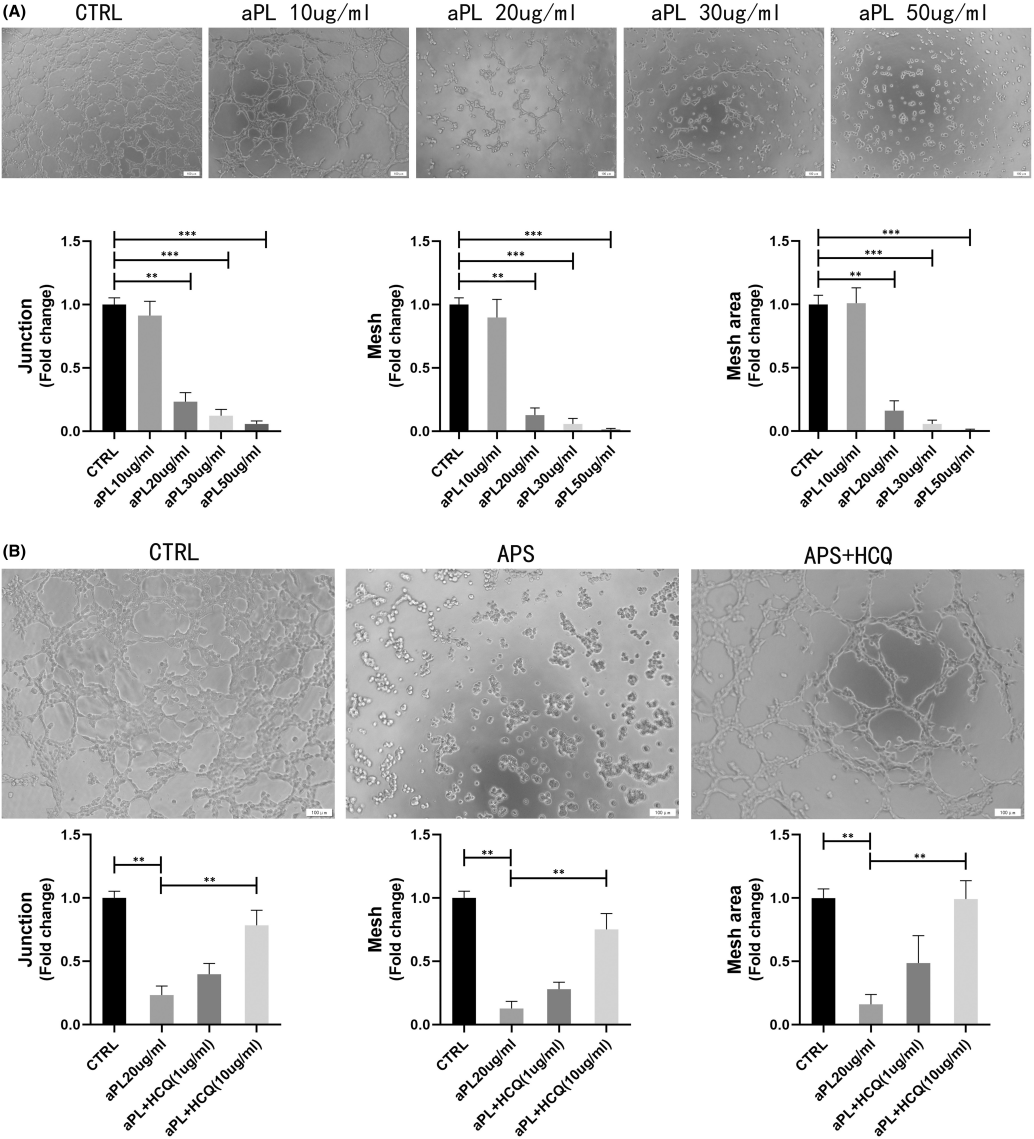
Effect of HCQ on OAPS induced detrimental effects on the ability of tube formation of trophoblasts. Images of tube formation assay at 6 h. (A) HTR8 was incubated with no treatment or aPL at 10, 20, 30, 50 μg/ml, and Images of tube formation assay were measured at 6 h. Photographed at 10× objective. Values represent mean ± SEM. The number of tube junctions, meshes and mesh area was measured. The tube formation ability is shown as a percentage of the no treatment control. (B) HTR8 was incubated with no treatment or aPL at 20 μg/ml, with or without HCQ at 1 μg/ml and 10 μg/ml. Photographed at 10× objective. Values are shown as mean ± SEM. The number of tube junctions, meshes and mesh area was measured. The tube formation ability is shown as a percentage of the no treatment control. Data are from three independent experiments

OAPS is mediated by antiphospholipid antibodies and affects the placental structure and function.^8^ To assess the effect of trophoblast cells on spiral artery remodelling, a tube formation experiment was performed. In our study, exposure to aPL made it almost impossible to form tubules, which means that aPL affects the HTR‐8 tube formation ability. With an increasing aPL dose, the degree of tube formation gradually decreased. The adverse effects of APS on placental vascular remodelling became increasingly serious with increasing doses (Figure 2A). Treatment with HCQ (10 μg/ml, not 1 μg/ml) improved this effect. We found that the decrease in these indicators (junction, mesh, mesh area) caused by aPL was improved by the addition of HCQ therapy. For the junction and mesh area, HCQ can improve the adverse effects of APS and can have a significant effect but cannot completely reverse its effect and restore it to the normal control level. Interestingly, HCQ was particularly effective in improving the mesh area, reaching 99.1% of the control group (Figure 2B).

### 3.4 HCQ reverses the adverse effects of aPL on trophoblast invasion and migration

**FIGURE 3:**
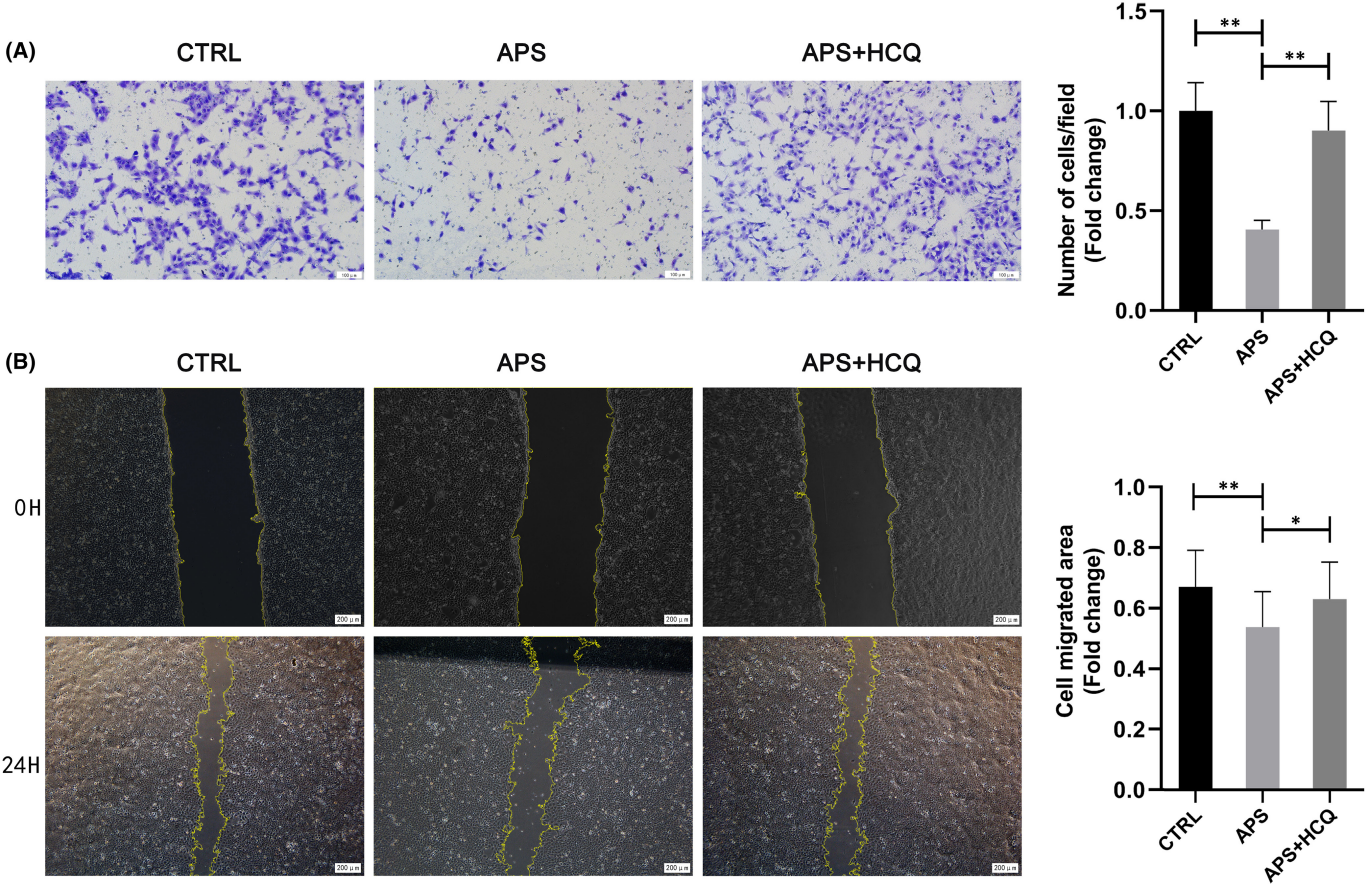
Effect of HCQ on OAPS induced detrimental effects on the ability of invasion and migration of trophoblasts. HTR8 cells were incubated with aPL (20 μg/ml) in the presence of HCQ (10 μg/ml) or not. (A) Images of Transwell invasion assays, measured after 24 h. Photographed at 10× objective. After 24 h, the number of transmembrane cells were measured. The invasion ability is shown as a percentage of the no treatment control. Values are shown as mean ± SEM. (B) Images of wound‐healing assays, measured at 0 h and 24 h. Photographed at 4× objective. The number of cell migration was measured. The migration ability is shown as a percentage of the no treatment control. Values are shown as mean ± SEM. Data are from three independent experiments. *p < .05; **p < .005

The human first‐trimester extravillous trophoblast cell line HTR‐8/SVneo was used to explore the function of HCQ in aPL‐induced placental injury. Migration and invasion are important for trophoblasts in placental development. In the Transwell assay, HTR‐8/SVneo cell invasion was more inhibited by antiphospholipid antibodies compared with the control; incubation with aPL significantly decreased the number of invading HTR‐8/SVneo cells from the upper chamber to the lower chamber. Compared with the control, the number of invaded cells decreased to less than 50% in the APS group, while the adverse effect was significantly reversed in the HCQ group. HCQ increased the percentage of APS passing through the cells from 40.5% to 90.3%, as compared to the control group (Figure 3A).

The migration of trophoblast cells also plays an important role in the normal formation of placental villi. The wound‐healing assays showed that aPL could also reduce the cell migration area. Compared with the control group, in which the scratch area increased by 67.7% at 24 h, the APS group only increased by 54.3%. After treatment with HCQ, it increased to 63.7%, which was not significantly different from the control group (Figure 3B). HCQ as a therapeutic agent reversed the aPL‐induced changes in HTR‐8/SVneo cell invasion and migration.

### 3.5 The OAPS mouse model was successfully constructed by means of the aPL injection

**FIGURE 4:**
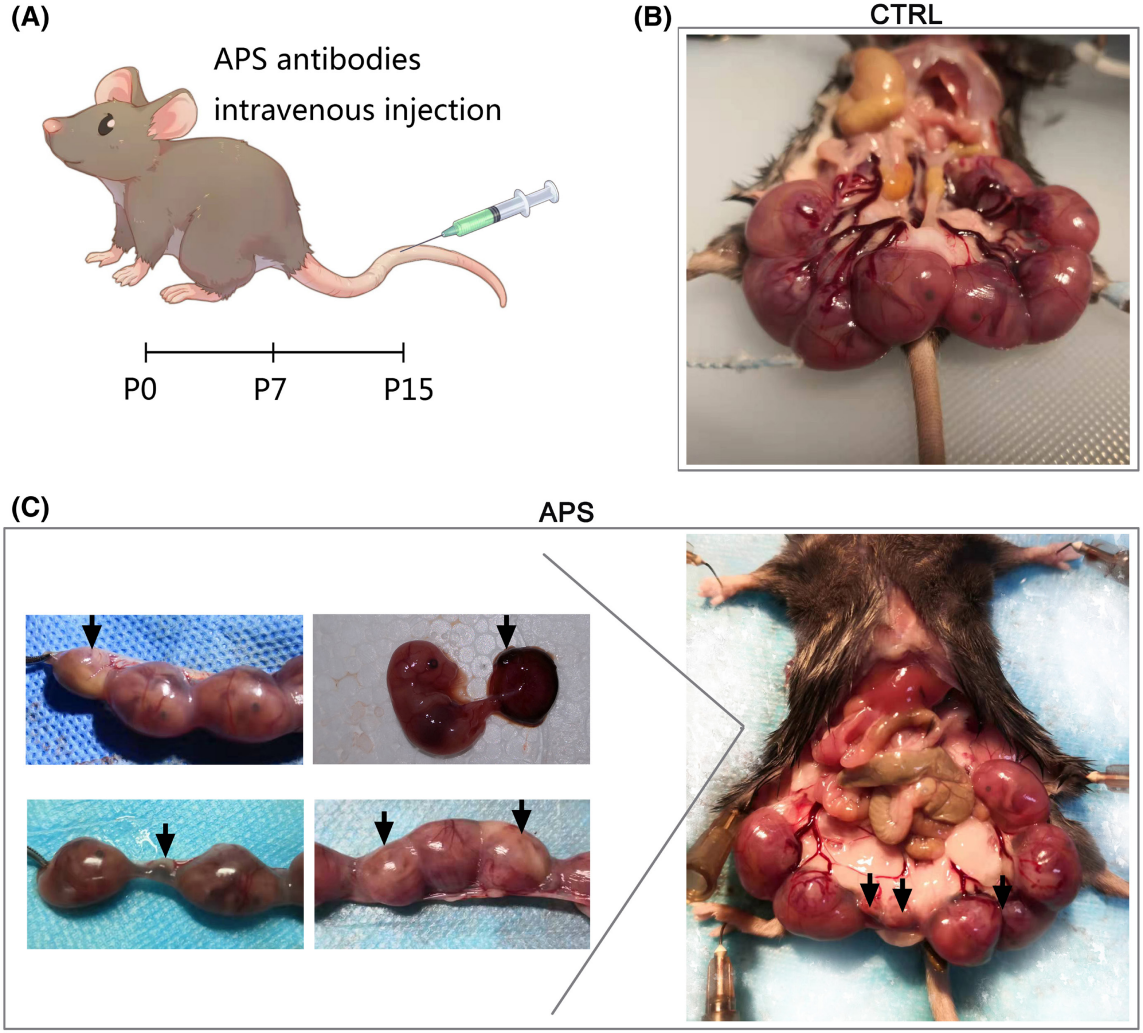
The mouse model of OAPS was obtained by injection of aPL, presenting as fetal loss and placenta abnormalities. (A) Schematic diagram of making OAPS mouse model. Administration by tail vein injection in mice to construction the OAPS mouse model. (B) Representative photographs of uterus morphology from normal mouse on P15. (C) Representative photographs of uterus morphology from OAPS mouse on P15. Injected with aPL during pregnancy to induce foetal loss. Abortion, stillbirth, growth restriction, and placental hematoma were observed in OAPS mouse models, as the arrow points out. Data are representative of observations in 5 mice per group

An OAPS model was constructed to determine the pathogenicity of antiphospholipid antibodies in vivo. APS mouse models were established by an intravenous injection of aPL antibodies (Figure 4A,B). It is important to note that OAPS mainly presents as pathological pregnancy rather than vascular thrombosis.^13^ Compared with passive immunization, a tail vein injection allows antibodies to be present in the circulatory system and directly affects the maternal–foetal interface, which directly affects placenta development.

**FIGURE 5:**
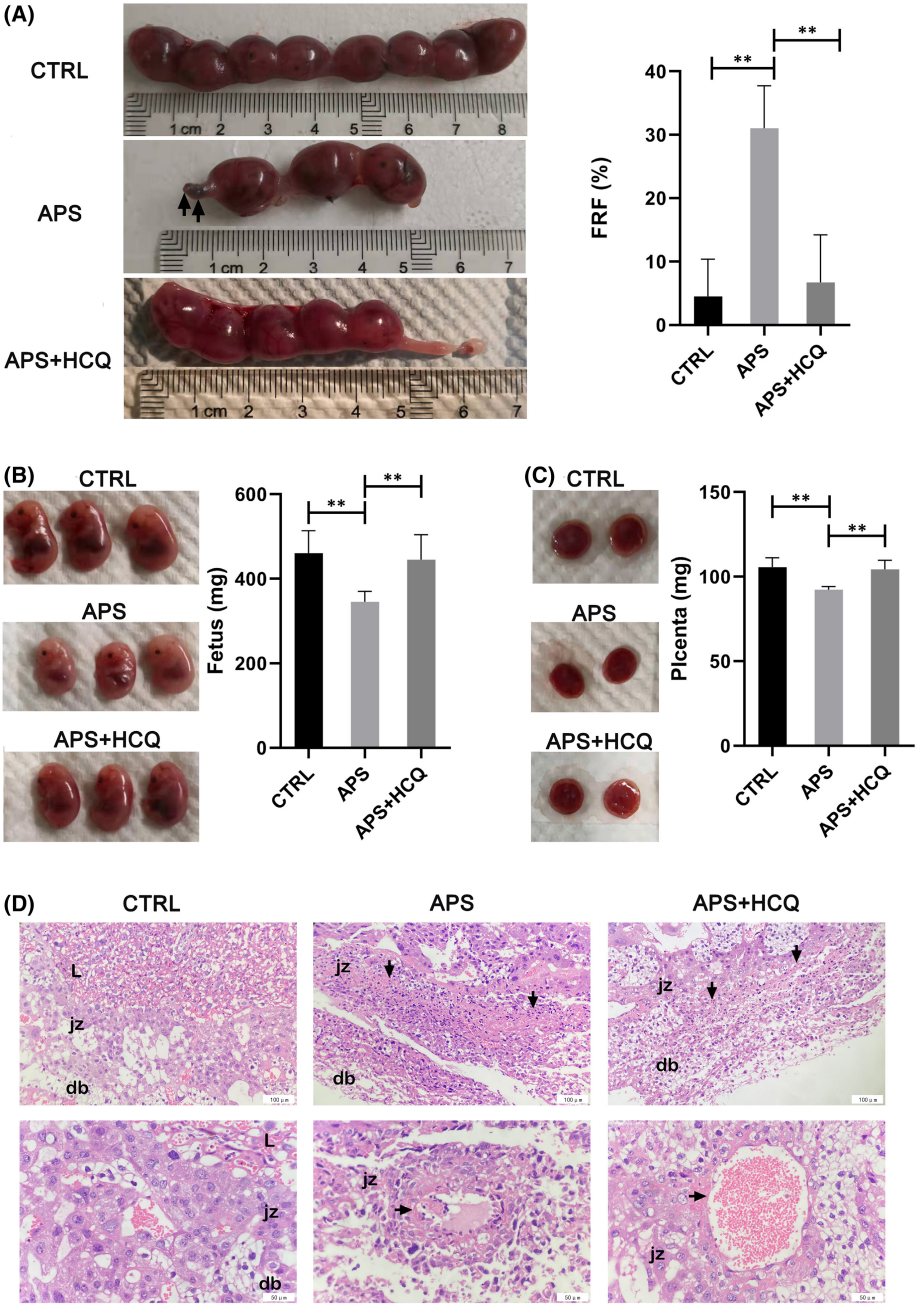
HCQ reduced fetal absorption rate and placenta abnormalities in OAPS. The pregnant mice were treated with physiological saline, aPL, aPL + HCQ. (A) Representative photographs of uterus morphology on P15. FRF was calculated as the number of resorptions divided by the total number of fetuses (resorptions plus viable). Values are shown as mean ± SEM. (B) Representative photographs of fetus. The weight of fetus was compared among all groups. Values are shown as mean ± SEM. (C) Representative photographs of placenta. The weight of placental was compared among all groups. Values are shown as mean ± SEM. (D) Representative photographs of histological analysis of the placentas. The arrow points out the necrosis area and diseased blood vessel in the labyrinth (L) and the junctional zone (jz), and decidua basalis(db). Pathological foci were obtained at 20× objective. Vascular lesions were obtained at 40× objective

In our APS model, approximately 31% of the embryos did not survive 15 days after the start of pregnancy (Figure 5A, Table 3). The surviving foetus could have growth restriction, stillbirth and intrauterine distress, which were consistent with the clinical manifestations of OAPS. Visible bleeding areas even appeared in part of the placenta in a few APS models (Figure 4C). Compared with the control, the placentas were smaller, and the average foetal weight was decreased in the OAPS model mice, suggesting that antibodies have important effects on the maternal–foetal interface formation, material transport and nutrient exchange.

**TABLE 3:** Therapeutic effects of HCQ in animal models of APS

	FRF (%)	Foetus (mg)	Placenta (mg)	Weight difference (g)	No pregnant proportion
CRTL	4.5 ± 5.6	460.5 ± 49.4	105.6 ± 5.2	10.1 ± 1.5	11.80%
APS	31 ± 6.4	345.8 ± 22.5	92.3 ± 1.7	10.4 ± 1.5	32.20%
APS + HCQ	6.7 ± 7.1	445.3 ± 54.6	104.3 ± 4.9	9.8 ± 0.9	18.20%

*Notes*: FRF was calculated as the number of resorptions divided by the total number of foetuses (resorptions plus viable).

Abbreviation: FRF, foetal resorption frequency.

The foetal resorption frequency increased compared with other APS models in which antibodies were given by an intraperitoneal injection or by a subcutaneous injection.^16^, ^33^ Additionally, compared with the patient's serum antibodies, which are difficult to purify due to their wide variety, the model formed by prepared antibodies had a higher abortion rate and was more stable.

### 3.6 HCQ prevented foetal death and placental abnormalities in OAPS

HCQ is effective in the treatment of OAPS, which is manifested as a reduced embryo absorption rate and an increased foetal and placental weight (Figure 5B,C). The dosage of HCQ that was used was based on the clinical medication for autoimmune diseases (the minimum effective dose should be used for maintenance and should not exceed 6.5 mg/kg/day or 0.4 g/day). HCQ effectively reversed adverse pregnancy outcomes. Compared with the control mice, the HCQ‐treated mice had normal foetal weight and placental size and had few pregnancy complications.

It is worth noting that despite the significant difference in the embryo absorption rate, the weight difference between P15 and P0 of each group of pregnant mice was not statistically significant. However, there were significant differences between the groups of mice that were not pregnant after the vaginal plug. Compared with the controls, aPL significantly reduced the ratio of successful pregnancy after the vaginal plug, while HCQ treatment failed to restore the normal level (Table 3). Whether this means that HCQ has a longer onset time than antiphospholipid antibodies or that HCQ mainly targets the developmental stage after placenta formation is worthy of further study.

### 3.7 HCQ alleviates the pathological reactions in the placentas of OAPS mice

To judge the effect of APS on placental cell tissue and the therapeutic effect of HCQ, we evaluated pathological tissue sections on mouse placentas. Compared with normal placentas, there were some punctate or banded cell necrosis lesions near the decidua basalis of the placentas from OAPS mice (Figure 5D). Lesions appear as lumps/cords/sheets. The necrotic area of the placenta in the OAPS group was larger and extended into a wider strip, while the necrotic area of the placenta in the HCQ group was greatly reduced, and there was still some small cord‐like necrosis. At the same time, other pathological reactions, including calcium deposition, fibrinoid degeneration, eosinophilia, vasodilation, failure of vascular remodelling and thrombosis, were more or less present in the placentas of OAPS mice. In the OAPS model, the damage of placental blood vessels was also more obvious. The cells in the vessel wall are degenerated and shed, or the vessel wall thickens to form necrosis foci, and in severe cases, the lumen collapses. We found that in the pathology of OAPS mouse placentas, many lesions often appeared near the decidua of the maternal surface of the placenta. This indicates that the junctional zone (jz) between the decidua basalis(db) and the labyrinth (L) is an area of high incidence of lesions. This also confirmed that aPL has adverse effects on the formation of the trophoblast villi and even in the stable implantation of the maternal–foetal interface. This is consistent with our in vitro experiments.

After HCQ treatment, these conditions were effectively alleviated. Although there were still some small necrotic foci, the number and area were significantly reduced. The pathological results showed that HCQ plays an important role in placental development and vascular remodelling and improves the function of placental trophoblast cells.

## 4 DISCUSSION

Antiphospholipid antibodies cause placental‐mediated pregnancy complications, leading to recurrent abortion, preeclampsia or stillbirth.^8^, ^34^ Current research suggests that aPLs, especially anti‐β2GPI Ab, affect the invasion of trophoblast cells, causing superficial implantation of the placenta and placental insufficiency.^35^, ^36^ Placental formation includes not only an invasion of trophoblasts but also the spiral artery remodelling process, forming a maternal–foetal interface for material exchange.^37^ The effect of aPLs on the overall process of placenta formation is still lacking. Our study demonstrated that aPL not only affects the invasive function of trophoblast cells but also affects vascular remodelling, placental formation and development. And aPL causes pathological damage at the maternal–foetal interface. This can be corroborated in in vivo and in vitro experiments.

APS patients are often complicated with other immune diseases, and IgG antibodies in the body are diverse and highly heterogeneous.^2^, ^38^ Compared with IgG extracted from the patients' blood, we used anti‐β2GPI Ab, which has a single component and a strong repeatability, and there is no interference of specimen heterogeneity. Regardless of active immunization or passive immunization, OAPS models have difficulty in maintaining stable plasma concentrations with time, reduces individual differences and are indistinguishable from the APS model.^39^, ^40^, ^41^ Studies have shown that antibodies are deposited quickly in mice, and the blood concentrations are reduced.^16^, ^42^ Our study can ensure blood drug concentrations to the maximum extent, similar to HCQ and aPL medication. Therefore, we can simulate the clinical findings in patients as much as possible.

**FIGURE 6:**
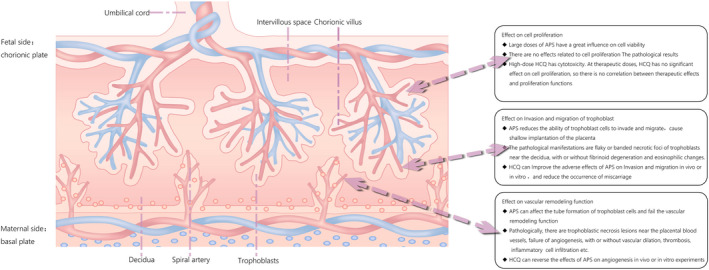
Placental pattern diagram of OAPS treated by HCQ

HCQ has been applied as a clinical treatment in pregnant women with APS, but the therapeutic mechanism is unclear.^43^ Some studies have shown that HCQ can inhibit aPLs binding to trophoblast cells.^27^, ^29^ On the basis of previous research, our experiment improved the protective mechanism of HCQ on placental trophoblasts in many aspects (Figure 6). Our study confirmed that HCQ could improve the invasion function of trophoblast cells, which was consistent with other studies. At the same time, we found that HCQ can also improve the damage of aPL to trophoblast cell formation, protect the formation of the placenta and reduce the miscarriage rate and pregnancy complications. HCQ is beneficial for placental formation and development and can improve the adverse foetal outcomes caused by APS, such as stillbirth and foetal growth restriction. At present, HCQ is used for refractory APS or empirical medication, which may underestimate its therapeutic potential.^23^, ^32^

Thrombosis and inflammation are thought to cause placental dysplasia in APS patients.^35^ Studies have shown that complement activation and neutrophil activation can cause foetal injury in OAPS patients.^14^, ^44^, ^45^ For the treatment of autoimmune diseases, current studies have hypothesized that HCQ can reduce the release of inflammatory factors mainly due to its anti‐inflammatory effect.^46^, ^47^, ^48^ Some studies have speculated, based on experimental results, that HCQ may be involved in improving APS‐induced energy metabolism disorders in placental and foetal mice, as well as decreasing neuroprotection and loss of chemical transmitters.^14^, ^49^, ^50^ The results of our OAPS animal models showed that trophoblasts invading the decidual area often had small necrotic foci, which could be accompanied by inflammatory cell infiltration and the formation of calcification foci. Therefore, we speculate that the inflammatory response, oxidative stress and metabolic function pathways may be the key to the treatment of placental injury in OAPS patients by HCQ. We will continue to look for the mechanism of HCQ on OAPS from these directions.

There are still some debates about the security of HCQ during pregnancy. In some views, HCQ has adverse effects, using it should increase the vigilance. There is a study that in autoimmune rheumatic disorders, using HCQ in first trimester would slightly increases the risk of malformations.^51^ HCQ has side effect on the eyes and cause heart arrhythmia in patient, and has a rare risk for genotoxic, that cannot be entirely ruled out.^52^, ^53^ Others believe using HCQ is safe during pregnancy. HCQ during pregnancy has not been shown to increase the risk of prematurity, low birth weight or major congenital malformations.^53^, ^54^, ^55^ In 2019, European League Against Rheumatism recommended that HCQ could be included in the addition of treatment strategies of OAPS.^4^ HCQ doses applied to OAPS are small (typically 0.2–0.4 g qd), and we have been using HCQ in obstetrical clinics for about five years. In consideration of side effects, liver function, kidney function and eyes are closely monitored during medication. So far, we have not seen any OAPS patients with complications from taking HCQ, nor teratogenic foetuses. Given that OAPS is not a disease with a high incidence, and some atypical cases may have confounding factors, expanding the number of cases included in the study makes the conclusions more rigorous. We will improve this aspect in future studies.

Although we have performed various experiments on the effect of HCQ on trophoblasts in OAPS, the molecular mechanism of HCQ has not been thoroughly investigated. To conduct further research, we are carrying out relevant RNA‐seq, ATAC‐seq and ChIP‐seq to systematically explore the transcriptional regulation mechanism of HCQ function, and relevant work will be published in subsequent studies. Our team has confirmed that platelet‐derived microparticles (PMPs) have adverse effects in OAPS, by affecting the function of trophoblast cells and endothelial cells.^56^ The effect of HCQ on metabolism and transmitter secretion requires us to think about whether PMPs is one of its therapeutic targets. This is our next research direction.

There are studies that suggest a ApoER2 is associated with the pathogenesis of APS.^57^, ^58^ By binding apoER2 to β2GPI, aPL plays a role in inducing foetal loss and IUGR. This is consistent with our conclusion that aPL damages the placenta and causes adverse pregnancy outcomes. Also, the study suggest apoER2 is abundantly expressed in the labyrinth of the mouse placenta. Could this be a potential therapeutic target for HCQ? We will continue to study it.

Recently, there has been interest in the potential efficacy of HCQ against coronavirus disease 2019 (COVID‐19) and sparked a wide‐ranging discussion about HCQ.^59^, ^60^, ^61^ This makes the anti‐inflammatory and antiviral effects of HCQ more important. Our work suggests that placental injury in OAPS can be partially reversed by HCQ, which can protect the placental function and reduce complications. The results of in vivo and in vitro experiments are consistent and are mutually supporting.

Autoimmune diseases, including APS, have a significant impact on maternal and foetal health. The placenta, as a complex organ, is vulnerable to antibody attack, and multidimensional damage can occur. The mechanism of HCQ can provide new ideas and treatment options for placental diseases caused by immune dysfunction.

## AUTHOR CONTRIBUTIONS

**Jing Liu:** Data curation (equal); investigation (lead); methodology (equal); writing – original draft (lead). **Liting Zhang:** Formal analysis (equal); investigation (supporting); software (supporting). **Yijia Tian:** Investigation (supporting); software (supporting); validation (equal). **Shuting Wan:** Investigation (supporting); validation (equal). **Min Hu:** Data curation (equal). **Shasha Song:** Methodology (equal); validation (equal). **Meihua Zhang:** Visualization (equal). **Qian Zhou:** Formal analysis (equal). **Yu Xia:** Conceptualization (equal); project administration (lead); resources (supporting); supervision (equal); writing – review and editing (equal). **Xietong Wang:** Conceptualization (equal); project administration (supporting); resources (lead); supervision (equal).

## CONFLICT OF INTEREST

The authors confirm that there are no conflicts of interest.

## Supporting information


Figure S1

Figure S2

## Data Availability

The data that support the findings of this study are available from the corresponding author upon reasonable request.
