# Supplementary figures and images for: Protection by hydroxychloroquine prevents placental injury in obstetric antiphospholipid syndrome
Source: J Cell Mol Med. 2022 Jun 29;26(15):4357–70. doi: 10.1111/jcmm.17459 (PMC9344817; doi:10.1111/jcmm.17459)

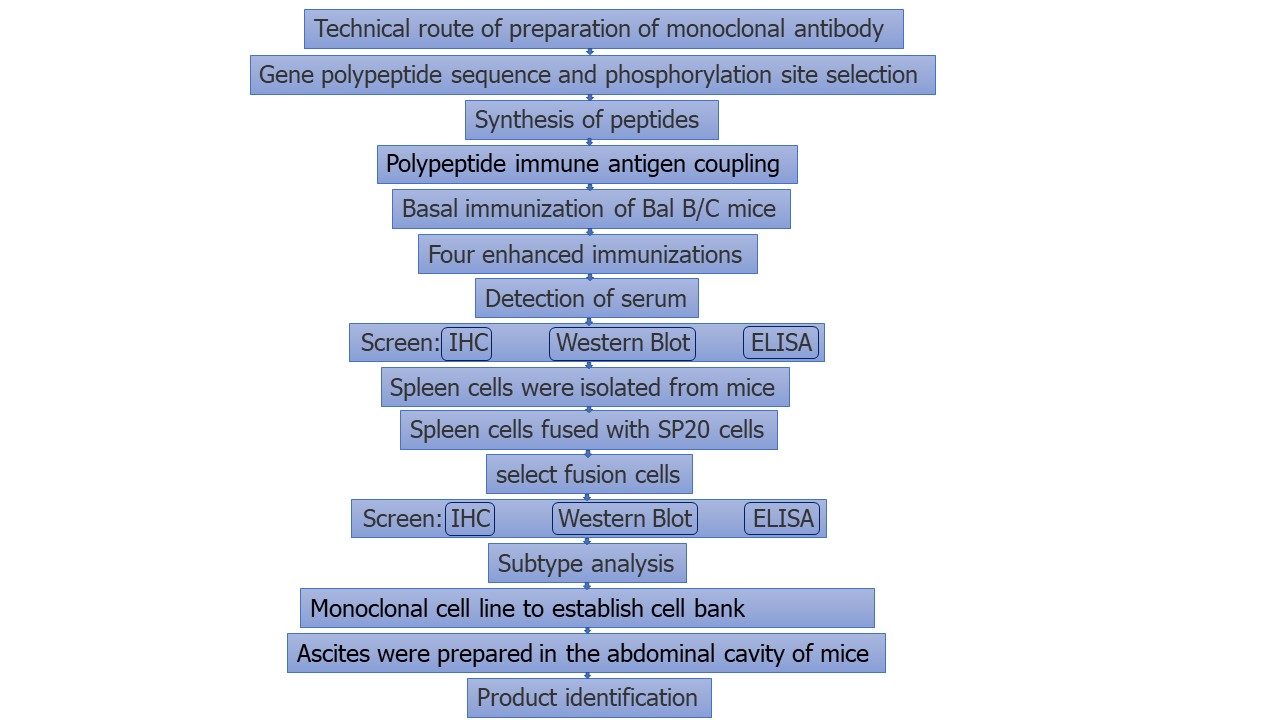

Supplement: Supplementary file 1 — Figure S1 [file JCMM-26-4357-s002.jpg]

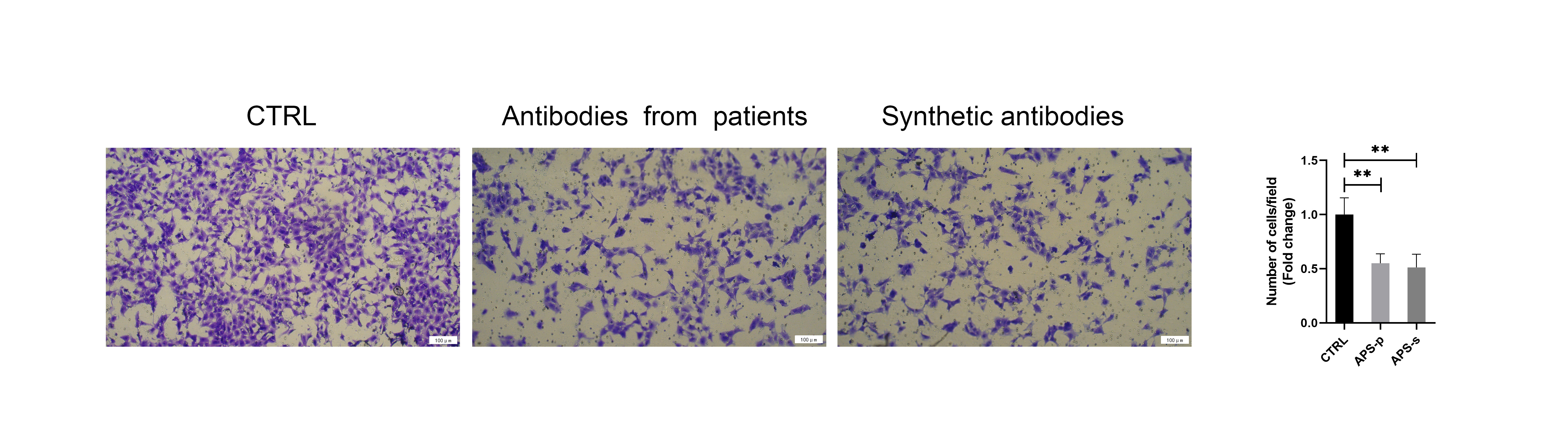

Supplement: Supplementary file 2 — Figure S2 [file JCMM-26-4357-s001.jpg]
